# A Psychometric Evaluation of the Family Collaboration Scale and an Investigation of How the Close Family of Frail Older Patients Perceive the Collaboration with Healthcare Professionals on Acute Medical Wards at Hospitals in Sweden

**DOI:** 10.3390/healthcare10030478

**Published:** 2022-03-03

**Authors:** Gerd Ahlström, Eva Björkman, Lars-Olov Lundqvist

**Affiliations:** 1 Department of Health Sciences, Faculty of Medicine, Lund University, 221 00 Lund, Sweden; 2 Department of Care Science, Faculty of Health and Society, Malmö University, 205 06 Malmö, Sweden; eva.bjorkman@mau.se; 3 University Health Care Research Center, Faculty of Medicine and Health, Örebro University, 702 81 Örebro, Sweden; lars-olov.lundqvist@regionorebrolan.se

**Keywords:** collaborative care, factor analyses, psychometric evaluation, frailty, elderly, hospital care, in-patient care, next of kin, significant others, relatives

## Abstract

The inclusion of family members in the acute care of older persons with complex needs results in better coordination of care and reduces the frequency and/or duration of rehospitalisation. Therefore, healthcare professionals need a tool to assess the collaboration with family members on acute hospital wards. The aims were to test the psychometric properties of the Swedish version of the Family Collaboration Scale (FCS), to investigate family members’ perception of collaboration with healthcare professionals on acute medical wards in Sweden and to compare the data with the corresponding Danish results. Three hundred and sixty family members of frail patients aged 65 or older from 13 acute medical wards answered the FCS questionnaire. In addition to descriptive statistics, psychometric methods were applied. The internal consistency of the Swedish version of the FCS was excellent, and confirmatory factor analysis revealed that its factor structure was equivalent to that of the original Danish FCS. The respondents’ ratings indicated better perceived collaboration than in the Danish case. Older age than 60 was associated with worse collaboration with healthcare professionals regarding *Influence on discharge* than younger respondents. Those with compulsory and health or nursing education showed better collaboration. The Swedish version of the FCS should be further evaluated for its retest reliability and as an outcome measure in intervention studies.

## 1. Introduction

For many frail older people, home care would not work without the contribution of family members as informal caregivers [1], and many researchers have predicted an increasing amount of informal caregiving in the future in societies with an ageing pattern similar to that of Sweden [2,3]. Of relevance in this connection is the broad international use of the policy term “ageing in place”, whereby older persons shall live and receive care in their home for as long as possible; it is a policy favoured both by policy makers and by many older people themselves [4,5,6]. Furthermore, people’s greater life expectancy means that there are an increased number of older people living with two or more chronic morbidities, and almost all chronic conditions are strongly related to ageing [7,8]. A review shows a 55–98% prevalence of multimorbidity in persons aged 65+ [8], and a Swedish population-based study showed a 55% prevalence among people aged 77–100 [9]. Multimorbidity affects not only the older people themselves but also the family members and the healthcare system, since it has been associated with poor quality of life and mental health, impaired functional ability and increased healthcare consumption financed by society [8,10,11]. A survey of 16 countries in Europe found an average of 37% multimorbidity among people 50 years and older and a strong association between multimorbidity and increasing age. Older people with multimorbidity compared to those without were hospitalised 49% more frequently in the previous year, made 35% more hospital visits and stayed in the hospital 49% longer [11].

The role of family members is substantial for frail older people with complex care needs, irrespective of how the particular country’s elderly care and healthcare are organised and financed [1]. Family members commonly feel a sense of responsibility and duty with regard to supporting the older person with informal care [12,13], and their commitment includes coordinating the multifaceted health and elderly care systems and negotiating treatment and support [13]. However, family members have often become frustrated by the difficulties that emerge when coordinating healthcare, and some question whether they have enough energy to continue. When older people need hospital care, family members can find it professional but unpredictable and inaccessible, without genuine interaction between themselves and healthcare professionals. Encounters with healthcare professionals can appear impersonal, sometimes demeaning, and insufficiently focused on the family member’s concerns [12]; this can, in turn, be due to overloaded healthcare professionals who are reluctant to suffer from further pressure [14].

A review of qualitative studies on the close family of older people admitted to the hospital found that the family members reported better quality and continuity in acute care when healthcare professionals applied relationship-centredness and a shared decision-making approach [15]. Another review with meta-analysis showed that the inclusion of family members in the discharge planning process meant reduced hospital readmissions, shorter rehospitalisation and lower post-discharge costs [16]. In their review, Park and colleagues [17] found that family-centred care interventions reduced the intensity of stress, anxiety and depression and increased satisfaction with the relationship with healthcare professionals. 

In Sweden, all patients are entitled to participate in decision making regarding their care and treatment, which, in the case of a frail older person, means the involvement of family members [18,19]. Such involvement requires the collaboration of healthcare professionals, who, in order to ensure quality of care, need information about the quality of their collaboration with family members. To meet this need, the Family Collaboration Scale (FCS) [20,21] was developed to assess family members’ perception of collaboration with nurses on acute hospital wards. Previous studies using the FCS have shown that family caregiver characteristics such as age and cohabitation with the patient are positively associated with the perception of better collaboration. In addition, a higher frequency of contact with nurses was associated with the perception of better collaboration [22]. There are validated versions of the FCS in Dutch [23] and Norwegian [24], but there is as yet none in Swedish. Against this background, the aims were to test the psychometric properties of the Swedish version of the Family Collaboration Scale (FCS), to investigate family members’ perception of collaboration with healthcare professionals on acute medical wards in Sweden and to compare the data with the corresponding Danish results.

## 2. Materials and Methods

### 2.1. Design

The study used a cross-sectional questionnaire design, and data were collected from five hospitals in southern Sweden using the Swedish version of the Family Collaboration Scale. 

### 2.2. Recruitment and Description of Participants

The study population consisted of the close family members of patients aged 65 or older with at least one chronic disease besides the health condition that had recently necessitated in-patient care on an acute medical ward. They were recruited consecutively from 13 acute medical wards at 5 public hospitals in the south of Sweden. Two of these were university hospitals, and three were rural hospitals. The hospitals were strategically selected in order to include facilities of different sizes and individuals with different socio-economic characteristics in the study sample. Inclusion criteria were that the family member should be the one who provided most of the care and support for the older person at home and that he or she could speak and understand Swedish. The recruitment of family members was performed by means of asking the patients. 

Four hundred and twenty-six family members were identified. Thirty-two of them refrained from participation, and the remaining three hundred and ninety-four received the questionnaire by mail. However, a further 22 refrained from participation after a reminder by phone. The reasons given for declining participation were fatigue, an overload of caregiving tasks after the older person’s discharge, emotional exhaustion and grief in relation to the recent death of the older person and, in a couple of cases, not being sufficiently involved in the older person’s life after hospitalisation. Thus, 372 family members completed and returned the questionnaire. Twelve of the completed questionnaires were thereafter excluded because more than ten of the items were not answered. Thus, the analysis was based on the remaining 360, which was 85% of the original number sent out. The characteristics of the study group are shown in Table 1. Mean age was 62.7 years (SD 11.8, range 20–95 years). Most commonly, caregiving family members were adult children (60%), followed by spouses (30%). In total, one-third (34%) cohabited with the older patient. The level of education was relatively evenly distributed, with about a third of the sample within each of the three educational categories: compulsory school, *gymnasium* and university. About one-fifth had completed a health or nursing education, and half of the family members were working. 

### 2.3. The Family Collaboration Scale

The Family Collaboration Scale (FCS) assesses collaboration between family members and nurses [21]. The original Danish version of the FCS consisted of 77 items [20], which were later reduced to 42 [21]. Thus, the revised 42-item FCS covers five dimensions of collaboration: (1) *Influence on decisions* (10 items); (2) *Quality of contact with nurses* (9 items); (3) *Trust and its prerequisites* (11 items); (4) *Achieved information level* (5 items); and (5) *Influence on discharge* (7 items). Responses were given on either a 4-point or a 6-point Likert scale. Response alternatives for the 4-point scales were the following: to a large degree, to some degree, to a lesser degree and not at all (rated 1–4). For the 6-point scales, they were the following: always, very often, often, sometimes, seldom and never (rated 1–6). Thus, on these scales, 1 represents the highest level of collaboration, and 4 or 6 represents the lowest. In the case of seven items (30, 31, 32, 35, 40, 47 and 53), it was possible to respond “don’t know”, and in the case of three items (20, 37 and 38), it was possible to respond “not relevant”. The questionnaire included additional variables covering demography (9 items), family members’ caring activities (5 items) and their feeling of responsibility and experiences of providing care (5 items). At the end of the questionnaire, the respondents had the opportunity to write comments as free text [21]. 

Since the FCS has not previously been used in Sweden, it was translated from Danish into Swedish. This was performed in three steps. First, a translation was conducted by a Swedish-Danish researcher in collaboration with a person fluent in both languages. Second, the first and second authors refined and changed certain terms to bring the text more in line with Swedish conditions. For instance, the word “nurses” in a few items was changed to “healthcare professionals” in the Swedish version because family members were not always expected to distinguish between a nurse and other healthcare professionals in certain situations since healthcare in Sweden is often team-based. Third, face validity was tested by 10 family members, which resulted in a few clarifications of terminology. 

### 2.4. Data Collection

A contact person (a registered nurse) on each acute medical ward at the five hospitals included was informed about the study during a meeting with the researcher, and written material about the study was provided. The contact nurse gave an information sheet to older patients, who, at the same time, were given the opportunity to ask questions about the study. They were then asked if they wanted the family member to be included in the study. If they agreed, the nurse handed over contact information in the form of telephone numbers of family members to the researcher. Then, the researcher contacted each family member by telephone in order to give them information about the study and ask if they were interested in participating. After oral consent, the questionnaire, written information, a consent form and a prepaid return envelope were sent to family members who had agreed to participate. The questionnaire was completed by the family member after the patient’s discharge. 

### 2.5. Methods of Analysis

Descriptive statistics were conducted using the SPSS statistical software package version 25 (IBM Corp., Armonk, NY, USA). Because the FCS items have different scales, each item was linearly transformed into values from 0 to 100 to be expressed as a percentage of the maximum score, with 0 being the highest level of collaboration. Missing responses were imputed with the sample mean of that item.

Cronbach’s alpha [25] was used to assess the internal consistency of the FCS. The 0.70 criterion was used cut-off for adequate internal consistency [26]. Confirmatory factor analysis (CFA) using LISREL 8.8 [27] with generally weighted least squares estimation was performed on the asymptotic covariance matrices and the polychoric and polyserial correlation matrix obtained by means of the PRELIS program [28]. The parameters were estimated by the weighted least squares method using the asymptotic covariance matrix, as recommended by Jöreskog and Sörbom [27], because the measurement variables are ordinal.

CFA tested the tenability of the a priori proposed factor structure model based on the Danish FCS [21]: items 34, 35, 36, 37A, 37B, 38A, 38B, 40, 41 and 42 were assumed to represent dimension (1) *Influence on decisions*; items 44A, 44B, 45, 46, 47A, 47B, 48, 49A and 49B represented dimension (2) *Quality of contact with nurses*; items 22, 23, 24, 25, 27, 28, 29, 54A, 54B, 54C and 54D represented dimension (3) *Trust and its prerequisites*; items 20A, 20B, 20C, 20D and 43 represented dimension (4) *Achieved information level*; and items 21C, 32, 37C, 37D, 38C, 38D, and 51 represented dimension (5) *Influence on discharge*.

The adequacy of the model was evaluated using the Satorra–Bentler scaled chi-squared test (S-Bχ2) supplemented with the comparative fit index (CFI), the standardised root mean square residual (SRMR) and the root mean square of approximation (RMSEA). Values equal to or greater than 0.90 and 0.95 for the CFI, equal to or lower than 0.10 and 0.08 for the SRMR and lower than 0.08 and 0.05 for the RMSEA were considered to constitute an excellent level of goodness of fit [29,30,31]. 

The mean value, standard deviation and *t*-test for the five factor dimensions of the FCS in respect of different demographic variables were analysed. The influence of the demographic variables on the five domains of the FCS as well as the total collaboration score was evaluated with univariate regression analysis. A *p*-value equal to or less than 0.05 was considered statistically significant.

## 3. Results

There were 7.4% missing responses in the FCS, which were imputed with the sample mean of that item.

### 3.1. Factor Structure of the Swedish FCS

The CFA performed in this study on the model that represents the original FCS factor structure [21] showed a significant chi-square (S-Bχ2 = 2618.20, df = 809, *p* < 0.001), a CFI = 0.92, an SRMR = 0.090 and an RMSEA = 0.079 (90% CI = 0.076–0.083), indicating an adequate goodness of fit. 

All but item 43 showed significant loadings on the expected FCS dimension. In addition, although significant, items 21C and 32 received lower loadings than are generally regarded as adequate. Because these three items (43, 21C and 32) also showed poor estimates in the original Danish evaluation and it was suggested that they be deleted from future versions [21], a model without them was specified and reanalysed.

The revised model without these three items showed a significant chi-square (S-Bχ2 = 2291.60, df = 692, *p* < 0.001), a CFI = 0.93, an SRMR = 0.084 and an RMSEA = 0.081 (CI = 0.077–0.084), indicating a significantly improved fit according to chi-square (S-Bχ2 difference of 326.6 with 117 degrees of freedom with a *p* < 0.0001) and improved CFI and SRMR but a worse fit regarding RMSEA. The deletion of only item 43 resulted in a model with a significant chi-square (S-Bχ2 = 2529.48, df = 769, *p* < 0.001), a CFI = 0.92, an SRMR = 0.090 and an RMSEA = 0.080 (CI = 0.076–0.083), indicating a significantly improved fit according to chi-square (S-Bχ2 difference of 88.72 with 40 degrees of freedom less with a *p* < 0.0001) but no improvement in CFI, SRMR or RMSEA. Given the lack of distinct model improvement attainable by deleting any items, the original model was deemed adequate to explain the factor structure of the FCS. However, since the deletion of item 43 would substantially improve the internal consistency (from below adequate (0.69) to good (0.85)) of dimension (4) *Achieved information level*, item 43 was deleted from further analyses. Summary statistics of the final model as well as internal consistency of the total FCS and five subscales are presented in Table 2. 

### 3.2. Differences in FCS across Groups

Analyses of group differences (Table 3) revealed no significant gender-related differences. The only age group difference was observed on subscale (5) *Influence on discharge*, where respondents older than 60 perceived significantly worse collaboration than younger respondents. Respondents with a higher education level, such as *gymnasium* or university, perceived significantly worse collaboration (higher total FCS as well as higher FCS subscale ratings) than those with only a compulsory school education. On the other hand, those with a health or nursing education perceived significantly better collaboration (lower mean FCS total and lower ratings in (4) *Achieved information level* and (5) *Influence on discharge*) than those who had no health or nursing education. There was no significant difference in FCS ratings between those who were cohabiting with the frail older person and those who were not. 

### 3.3. Relationship between the Respondent’s Feelings and FCS Ratings

As shown in Table 4, univariate regression analysis showed no relationship between respondents’ feeling of being responsible for the older person’s wellbeing and FCS. However, feeling responsible for the older person’s care was related to better perceived collaboration, as indicated by lower ratings on (1) *Influence on decisions* and (5) *Influence on discharge*. That is, the more responsible the family member felt, the better they perceived their influence on decisions and on discharge. Feelings of powerlessness, guilt and insufficiency were all significantly related to higher FCS ratings, indicating worse perceived collaboration with the nurses on the wards. This was particularly related to the perception of (2) *Quality of contact with nurses*, (3) *Trust and its prerequisites* and, to some extent, (4) *Achieved information level*. 

### 3.4. Comparison with Corresponding Danish Results 

The Danish sample (*n* = 388) used for comparison in the present study involved exactly the same inclusion criteria and method of recruitment as our own sample. This is described in the study by Lindhardt and colleagues [21]. The differences in the background data were as follows: 10% more cohabiting family members (34% vs. 24%) and 7.3% percent more spouses (30.5% vs. 23.2%) in Sweden.

In the comparison with FCS scores for the corresponding sample of close family members of frail older patients on acute medical wards in Denmark [21], the total mean score and the mean scores of the subscales were higher (worse perceived collaboration) in the Danish sample than in the Swedish one, except for (5) *Influence on discharge* (Table 5). Despite the difference in magnitude, the ranking order of the five subscales was equal between the Swedish and Danish versions, with better perceived collaboration found for (3) *Trust and its prerequisites* and worse for (1) *Influence on decisions*. 

## 4. Discussion

This study presents the first results of testing and investigating with the Swedish version of the FCS. The following discussion is geared toward the three aims of the study: (1) to test the psychometric properties of the Swedish version of the FCS, (2) to investigate family members’ perception of collaboration with healthcare professionals on acute medical wards and (3) to make a comparison with corresponding Danish results. 

### 4.1. Psychometric Evaluation

With item number 43 excluded, as also suggested on the basis of the Danish study [21], the CFA showed that data from Swedish respondents adequately fitted the factor structure of the original Danish FCS, supporting its construct validity and cross-cultural validity. The internal consistency was excellent for the total FCS and between adequate and good for the FCS subscales but somewhat lower than that observed for the Danish version [21]. Although the Swedish ratings indicated higher perceived collaboration than the Danish results, the ranking order of the five subscales was the same as in the Danish study [21], as was also the case in studies performed in Norway [24] and the Netherlands [23], which further supports the cross-cultural validity of the FCS. We suggest that the psychometric properties of the Swedish version be further evaluated through test-retest studies in repeated assessments and as an outcome measurement in intervention studies. This can be beneficial for healthcare professionals interested in the assessment of quality of care. 

### 4.2. Family Members’ Perception of Collaboration with Nurses

There were some differences between the results from the Danish and Swedish FCS with regard to the background variables. Contrary to the Danish situation [21], there were no differences related to gender in the Swedish results. However, a study and review by Jergermalm [2] showed that gender is highly relevant in research about informal caregiving. That study showed that women spent more time providing care than did men, whilst men provided more practical help. Healthcare professionals at the hospital can gain invaluable information about the patient from women who are heavily involved in providing informal care in the older person’s home.

Consistent with the Danish results [21], there were no age differences, except for older family members who reported worse quality of collaboration (higher ratings) in the case of *Influence on discharge*. Research shows that older family members are more often cohabitants with the patients and provide a lot of personal care [2]. These family members often have health problems of their own that can become obstacles to taking on increased responsibility for care after the patient’s acute hospital visit. However, previous research showed that family caregivers involved in discharge planning perceived pressure to undertake a greater level of responsibility for care, contributing to an increased feeling of having to bear a burden and increased stress. Sometimes, professionals have treated discharge planning as a routine task or as an opportunity to push through their own, often unspoken, ideas and suggestions for the family member [16,32,33]. Furthermore, the subscale *Influence on decisions* was ranked lowest, confirming family members’ dissatisfaction with their involvement in older persons’ care. Research has found that family members want to be in partnership in an endeavour to ensure that their relatives have a good life [12]. A systematic review of discharge planning identified several obstacles: nurses’ inadequate knowledge of patients’ activities, inability to define discharge planning and uncertainty over the timing of such planning (when to begin and when to implement it) [34]. This indicates that healthcare professionals in hospitals need to consider family members as partners and allow them to influence the conditions for patients’ discharge.

Respondents with more than compulsory education perceived worse collaboration than those with compulsory education only. One possible explanation is that the former had higher expectations [2]. 

In the Danish study [21], no significant difference regarding education or health or nursing education was observed. In our study, the opposite pattern was observed for health or nursing education, with better FCS ratings of collaboration among those with a health or nursing education. However, it is unknown whether these family members were more likely to appreciate that collaboration is a complex task because they also worked in healthcare. An additional background question for those who had health or nursing education could reveal whether they have dual roles.

The total mean score and the mean scores of the subscales show better collaboration in this study than in the Danish study [21], except for *Influence on discharge*. Furthermore, the highest rating of perceived collaboration was on the subscale *Trust and its prerequisites*. The results might be an expression of person-centred care, a policy and criterion for quality of care applied for more than a decade in Sweden [35]. Person-centred care implies that the patient is viewed as a person with resources and abilities. Such care is characterised by listening to what the person has to say and by partnership and agreement between the patient, family and healthcare professionals regarding interventions [35,36,37]. Since collaboration is part of person-centred care, the FCS should be tested as an outcome measure in the implementation of person-centredness in practice.

From the literature, it is evident that family members provide care as a natural, moral response, motivated by their love and compassion for the older person. Helping is often an obvious choice and connected with a feeling of responsibility [12,13]. The results of this study show (Table 4) that the feeling of responsibility for the older family member receiving the right service was associated with better collaboration ratings on the FCS regarding *Influence on decisions* and *Influence on discharge*. In contrast, feelings of powerlessness, guilt and insufficiency were related to worse perceived collaboration. The results on guilt and powerlessness are in line with the Danish ones [21], indicating that such feelings, together with a feeling of insufficiency, are highly relevant for family members’ perception of the quality of collaboration with healthcare professionals. There has been a lot of investment in the implementation of person-centred care in clinical practice [35], but this study indicates that further effort is required, with increased focus on the co-creation of healthcare involving patients, family members, healthcare professionals and managers.

### 4.3. Methodological Considerations

One strength of this study is that the FSC was developed from a theoretical framework of collaboration between family caregivers and hospital nurses [20]. The FCS was a 56-item scale before being revised by Lindhardt and colleagues [21]. Parallel to the Danish FCS is a version in Dutch revised as a 20-item scale, narrowing the focus and including only items on collaboration, evaluated using face and content validity [22,23]. In addition, the sample in the study based on the Norwegian version of the FCS was smaller (n = 147), with many drop-outs (34%), and the psychometric methods were different [24]. These differences limited the comparison of our results with the Dutch and Norwegian ones. Further evaluation of the revised Danish version with different samples and in different settings would strengthen the conclusions about the FCS’s psychometric properties.

The FCS was developed and tested in the metropolitan area of Denmark [20,21], which is close to southern Sweden, where this study was performed. The Danish and Swedish regions are connected by a bridge, and many people live on one side and work on the other. The Danish and Swedish languages are relatively similar, and citizens of one country can, after some experience, understand citizens of the other. However, there are slight differences in healthcare systems and cultures between the countries [3,38,39,40], which we had assumed would cause the FCS to have poorer psychometric properties in a Swedish healthcare context. However, the results of this study showed that the FCS also works well in Sweden, which can be seen as an expression of the Nordic model of welfare [38,39]. 

In spite of the similarity between the two countries, the translation of the Danish FCS into Swedish led to the replacement of the term “nurses” by “healthcare professionals” in a few items, which was a result of pre-testing with 10 family members and our own experience as researchers from interview studies with family members. Swedish hospital care is characterised by teamwork, which makes the term “healthcare professionals” more appropriate; at the same time, the change in terms should increase the reliability of the results. This rewording was also applied in the Norwegian version [24]. 

Two items in the FCS need further development. The broad background question on health or nursing education needs to be specified in order to investigate whether longer nursing education is more important in the results than shorter health and nursing education. The background question concerning gender also needs to be updated with terms for gender identities other than just male and female.

A weakness of this study derives from the fact that the ethical committee decided that the diagnoses of the older persons were not to be included in the questionnaire. The committee’s opinion was that the collection of this confidential information was unnecessary since this study was about family members’ collaboration with healthcare professionals on acute medical wards. However, research has suggested that it is not the diagnosis itself that has the most influence on family members’ collaboration with healthcare professionals but rather the unpredictability of the illness, the complexity of care and the challenge of communication [41]. These facts are unknown in the case of the present study, which needs to be borne in mind when it comes to the interpretation and generalisability of the results.

The drop-out rate among family members who were asked whether they were willing to participate was relatively low (5.6%, i.e., 22 of 394). However, the recruitment of the family members started by asking the patients. A weakness derives from the unknown attrition rate at this stage when the contact nurse asked the older person’s permission for the researcher to contact the family member. It is possible that, due to heavy workload, days off and sick leave, contact nurses were not consistent in filling out the drop-out list of the older persons who refused. However, we assessed this as a minor issue because the nurse was well-known to the older person, which should have contributed to the latter’s willingness to allow contact with the family member about participation in the study. This also needs to be taken into consideration regarding the generalisation of the results.

The selection of the hospitals was made strategically with the goal of including family members from different socio-economic backgrounds in the study sample. Although limited socio-demographic data were collected, the authors’ knowledge of the catchment areas of the hospitals was such that there was an assurance of variation. We consider this to be a strength in terms of generalising the results to the rest of Sweden and similar populations.

The cross-sectional design of this study using the FSC needs follow-up with repeated measurements over time and also tests to determine whether the scale sensitivity is acceptable for measuring changes in the results of interventions.

## 5. Conclusions and Implications

The results strongly support the contention that the Swedish version of the FCS is a valid and reliable questionnaire with psychometric properties comparable to those of the revised Danish FCS. Older age among family members was linked to worse collaboration with nurses regarding *Influence on discharge*, whilst having health or nursing education and lower general education were associated with better perceived collaboration. The Swedish family members rated perceived collaboration higher than did family members in the compared Danish study. Feelings of powerlessness, guilt and insufficiency, as well as lower-rated collaboration regarding *Influence on discharge*, indicate the need for increased investment in person-centred and co-created healthcare at hospitals. The FCS is a promising questionnaire, and further evaluation of the Swedish version should enable its introduction to measure the effectiveness of interventions for the improvement of collaboration between family members and healthcare professionals in a hospital setting.

## Figures and Tables

**Table 1:** Characteristics of respondents.

Background Variables	Total (*n* = 360) *n* (%)	Women (*n* = 229) *n* (%)	Men (*n* = 131) *n* (%)	Spouse (*n* = 110) *n* (%)	Adult Child (*n* = 215) *n* (%)	Others (*n* = 35) *n* (%)
Age (*n* = 359) 18–49 50–64 65–79 80+						
	43 (12) 164 (46) 127 (35) 25 (7)	36 (16) 102 (45) 81 (36) 9 (4)	7 (5) 62 (47) 46 (35) 16 (12)	0 (0) 14 (13) 77 (70) 19 (17)	36 (17) 145 (67) 34 (16) 0 (0)	7 (20) 6 (17) 16 (46) 6 (17)
Gender Women Men						
	229 (64) 131 (36)			69 (63) 41 (37)	141 (65) 75 (35)	20 (57) 15 (43)
Cohabiting with the older patient						
	124 (34)	75 (33)	49 (37)	110 (100)	10 (5)	5 (14)
					
School Education						
Compulsory school	120 (33)	77 (34)	43 (33)	58 (53)	46 (21)	16 (46)
Gymnasium	126 (35)	68 (30)	58 (44)	26 (24)	94 (44)	7 (20)
University degree	114 (32)	84 (36)	30 (23)	26 (24)	76 (35)	12 (34)
Health or nursing education	65 (18)	50 (22)	15 (11)	22 (17)	42 (19)	6 (17)
Working	185 (51)	121 (53)	64 (49)	21 (19)	159 (74)	5 (14)

**Table 2:** Summary statistics of the confirmatory factor analysis model of the Swedish FCS (*n* = 360).

	FCS Items	Loading	α	Mean	SD
	FCS Total		0.93	39.93	14.66
	1. Influence on decisions		0.87	68.35	20.26
34	Healthcare professionals asked what knowledge I had of my relative’s situation.	0.460		64.75	17.60
35	My knowledge was used by the healthcare professionals.	0.602		76.17	24.91
36	Healthcare professionals asked my views on decisions that had to be made.	0.657		84.13	24.15
37A	I was informed about decisions made about treatment.	0.833		63.21	36.64
37B	I was informed about decisions made about care.	0.833		69.83	35.11
38A	I had an influence on decisions made about treatment.	0.688		84.13	27.83
38B	I had an influence on decisions made about care.	0.684		84.88	26.87
40	Healthcare professionals and I were in agreement about what should happen to my relative.	0.713		54.52	31.55
41	I was satisfied with the influence I had.	0.705		48.43	35.16
42	Frequency of contact with healthcare professionals.	0.323		53.36	30.91
	2. Quality of contact with nurses		0.87	25.50	16.07
44A	It was easy to find a nurse (on the phone) who knew my relative.	0.502		33.98	22.20
44B	It was easy to find a nurse (in person) who knew my relative.	0.573		31.29	22.08
45	Healthcare professionals were obliging when I contacted them.	0.683		12.64	16.54
46	Healthcare professionals had time to talk to me.	0.741		23.73	22.80
47A	It was OK to express my feelings.	0.623		24.22	19.40
47B	It was OK to express criticism.	0.593		34.02	21.33
48	Healthcare professionals understood my situation as a relative.	0.694		26.00	26.72
49A	I am satisfied with the extent of contact with healthcare professionals.	0.807		22.71	26.39
49B	I am satisfied with the quality of contact with healthcare professionals.	0.776		20.94	25.31
	3. Trust and its prerequisites		0.79	21.66	13.19
22	Experiences of mistakes and insufficient care during this hospital stay.	0.561		21.89	13.09
23	Experiences of mistakes and insufficient care during prior hospital stays.	0.282		22.40	15.15
24	I trusted that my relative got the care she/he needed.	0.797		12.57	22.53
25	I had to make sure my relative got the care she/he needed.	0.531		17.40	28.39
27	Healthcare professionals treated patients with respect.	0.552		9.26	16.57
28	It was my impression that the healthcare professionals were too busy.	0.289		53.47	27.85
29	It was my impression that the healthcare professionals were competent.	0.673		8.99	16.81
54A	The physical environment had sufficient space.	0.471		17.51	25.51
54B	The physical environment was clean.	0.532		16.78	23.72
54C	The physical environment was tidy.	0.502		16.51	22.95
54D	The physical environment offered the possibility of privacy.	0.446		41.52	34.48
	4. Achieved information level		0.85	32.28	26.17
20A	I felt well informed about my relative’s illness.	0.846		26.46	31.17
20B	I felt well informed about my relative’s care needs.	0.852		27.64	31.18
20C	I felt well informed about the plans after discharge.	0.703		37.07	33.29
20D	I felt well informed about how best to help my relative in time to come.	0.628		37.96	30.87
	5. Influence on discharge		0.79	51.88	21.44
21C	Need for information/training about how best to help my relative.	0.198		60.01	27.40
32	The problem leading to the admission was solved.	0.259		25.44	28.52
37C	I was informed about decisions made about the discharge.	0.776		52.91	39.00
37D	I was informed about decisions made about arrangements after discharge.	0.756		55.43	37.64
38C	I had an influence on decisions made about the discharge.	0.619		76.58	33.10
38D	I had an influence on decisions made about arrangements after discharge.	0.743		63.74	34.41
51	I found the plans concerning the time after discharge acceptable.	0.509		29.04	33.61

**Table 3:** Mean (standard deviation) of FCS total and subscales by responder’s characteristics.

Variable	FCS Total	1. Influence on Decisions	2. Quality of Contact with Nurses	3. Trust and Its Prerequisites	4. Achieved Information Level	5. Influence on Discharge
Gender Women Men						
	40 (15)	68 (21)	26 (16)	22 (14)	33 (27)	51 (22)
	40 (14)	69 (20)	25 (16)	21 (13)	31 (25)	53 (21)
*p*-value	0.952	0.724	0.896	0.377	0.540	0.394
Age						
≤ 60	39 (15)	68 (21)	25 (16)	21 (14)	32 (27	49 (22)
>60	41 (13)	69 (19)	26 (17)	23 (12)	33 (25)	56 (20)
*p*-value	0.112	0.558	0.663	0.249	0.521	0.002
Education						
Compulsory school	35 (13)	64 (20)	21 (12)	17 (11)	27 (23)	46 (20)
Gymnasium and university	43 (15)	70 (20)	28 (17)	24 (14)	35 (28)	55 (22)
*p*-value	<0.001	0.008	<0.001	<0.001	0.002	<0.001
Health or nursing education						
No	41 (15)	69 (20)	25 (16)	21 (13)	34 (27)	53 (22)
Yes	36 (14)	64 (21)	27 (18)	23 (14)	23 (21)	45 (20)
*p*-value	0.033	0.078	0.428	0.270	<0.001	0.004
Cohabiting with the older person						
No	40 (14)	68 (20)	26 (16)	22 (12)	31 (24)	51 (22)
Yes	41 (16)	70 (20)	25 (16)	21 (15)	34 (29)	53 (21)
*p*-value	0.572	0.369	0.844	0.221	0.293	0.484

**Table 4:** FCS total and subscale results regressed on the family member’s feelings in relation to the older person.

Variable	FCS Total	1. Influence on Decisions	2. Quality of Contact with Nurses	3. Trust and Its Prerequisites	4. Achieved Information Level	5. Influence on Discharge
15 Responsibility wellbeing	−0.016	−0.053	−0.034	−0.021	0.028	−0.002
16 Responsibility service	−0.125 *	−0.157 **	−0.101	−0.014	−0.073	−0.105 *
17 Powerlessness	0.239 ***	0.083	0.235 ***	0.246 ***	0.254 ***	0.102
18 Guilt	0.136 **	−0.036	0.186 ***	0.207 ***	0.153 **	0.045
19 Feeling of insufficiency	0.108 *	−0.026	0.156 **	0.203 ***	0.096	0.036

Note. * *p* < 0.05, ** *p* < 0.01 and *** *p* < 0.001.

**Table 5:** Comparison between the Danish and the Swedish scores for the FCS total and subscales.

FCS Subscales	Rank	Danish ^1^ M (SD)	Swedish M (SD)	*p*
3. Trust and its prerequisites	1	33 (19)	22 (13)	<0.001
2. Quality of contact with nurses	2	42 (21)	26 (16)	<0.001
4. Achieved information level	3	54 (23)	32 (26)	<0.001
5. Influence on discharge	4	55 (22)	52 (21)	n.s.
1. Influence on decisions	5	74 (22)	68 (20)	<0.001
Total mean score		51 (16)	40 (15)	<0.001

^1^ From Lindhardt et al. [21].

## Data Availability

The datasets analysed during the study are available from the project leader G.A. upon written request and in accordance with ethical approval.
